# Targeting Vascular Endothelial Growth Factor in Oesophagogastric Cancer: A Review of Progress to Date and Immunotherapy Combination Strategies

**DOI:** 10.3389/fonc.2019.00618

**Published:** 2019-07-16

**Authors:** Oliver Butters, Kate Young, David Cunningham, Ian Chau, Naureen Starling

**Affiliations:** Gastrointestinal Unit, Royal Marsden Hospital, London, United Kingdom

**Keywords:** gastroesophageal cancer, vascular endothelial grow factor, immunotharapy, ramucirumab, biomarkers, bevacizumab, tyrosine kinasa inhibitor

## Abstract

In 2014, the survival benefits seen in REGARD and RAINBOW studies led the way for the regulatory approval of ramucirumab in the second line setting in oesophagogastric (OG) cancer. Trials of other drugs targeting the vascular endothelial growth factor (VEGF) pathway have met with mixed results but this remains an important pathway for evaluation in OG cancer. Perhaps the most interesting ongoing trials are those which target VEGF in combination with immunotherapy, which have a sound scientific rationale. Given the emerging role of immunotherapy in OG cancer, this is an important area of innovation. This review aims to outline targeting VEGF in OG cancer, the rationale behind the continued interest in this mechanism and possible future directions in combination with immunotherapy.

## Introduction

Oesophagogastric (OG) cancer consists of esophageal, gastro-esophageal junctional (GOJ), and gastric cancer and is associated with a poor prognosis. Gastric and esophageal cancers are the third and sixth leading causes of cancer related death worldwide with an estimated 723,000 and 400,000 deaths in 2012, respectively (1). A SEER cancer statistics review revealed an increase in 5 year survival in OG cancers from 1975 to 2014, from 15.2 to 32.1% in gastric cancers and 5.0–21.1% in esophageal cancers (2), although this continues to be poor and the median overall survival (mOS) remains less than a year. Histologically, OG cancers are divided in to adenocarcinoma and squamous cell carcinoma with most esophageal cancers (72%) and nearly all gastric cancers (96%) being adenocarcinoma (3).

## Current Treatment Paradigm for OG Cancer

**Figure 1:**
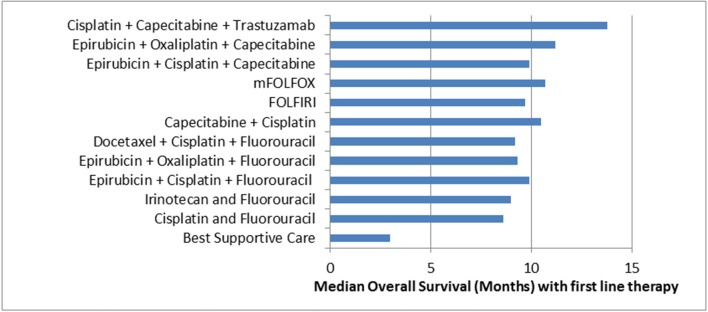
Median Overall Survival in Advanced OG Adenocarcinoma with selected first line therapy. Cisplatin + Capecitabine + Trastuzumab (5); Epirubicin + Oxaliplatin + Capecitabine (6); Epirubicin + Cisplatin + Capecitabine (6); mFOLFOX (7); FOLFIRI (8); Capecitabine + Cisplatin (9); Docetaxel + Cisplatin + Fluorouracil (10); Epirubicin + Oxaliplatin + Fluorouracil (6); Epirubicin + Cisplatin + Fluorouracil (6); Irinotecan + Fluorouracil (11); Cisplatin + Fluorouracil (10); Best Supportive Care (12).

Two thirds of Western patients present with advanced inoperable disease and for these patients, median overall survival is short (4). First line palliative chemotherapy for OG adenocarcinoma involves a platinum and fluoropyrimidine based doublet or triplet regimen with the addition of trastuzumab if Human Epidermal Growth Factor Receptor 2 (HER2) positive. Median overall survival is 3 months with best supportive care (BSC), less than a year with palliative chemotherapy and just over a year with the addition of trastuzumab in selected patients (Figure 1). Even with doublet and triplet chemotherapy, median survival of over a year is only achieved with the addition of the first biologic to be approved in this disease, trastuzumab. There is considerable geographical variation in survival, with improved survival in Japanese patients compared to western patients being well-documented (13–15).

Half of patients receiving first line chemotherapy can be expected to proceed on to second line chemotherapy on progression, although this figure varies considerably across the world, and there may be a role for sequential therapy for those who can tolerate it (16–18). Second-line chemotherapy with a taxane (docetaxel, paclitaxel) or irinotecan is recommended for patients who are of a good performance status (14–16, 19, 20). Such treatment has been shown to be superior to BSC by a number of studies with a 37% reduction in the risk of death (16, 21). However, the actual benefit remains limited, with mOS 3.8 months with BSC vs. 5.3 months with salvage chemotherapy and improved therapeutics are required (20). More recently, data has emerged to support the targeting of VEGF in the second line setting with the use of ramucirumab either as a single agent or in combination with paclitaxel. High level evidence for treatment in the third line setting is lacking but there is a role for immunotherapy emerging.

## Targeting Vascular Endothelial Growth Factor (VEGF)

Angiogenesis is mediated by the interaction between VEGFs and their tyrosine kinase receptors, VEGFRs. This mechanism can be targeted by monoclonal antibodies as well as by small molecule tyrosine kinase inhibitors. Inhibition of this pathway can be achieved at different levels using various mechanisms; with monoclonal antibodies to VEGFA or its receptor, with recombinant fusion protein to VEGF and with various multi-targeted tyrosine kinase inhibitors (TKIs). About half of gastric cancers overexpress VEGF and this is associated with a poor prognosis (22, 23).

As a hallmark of cancer, angiogenesis logically stands out as a potential target (24, 25). The hypothesis that malignant tumor growth is dependent upon angiogenesis has been demonstrated in multiple studies and this pathway has been successfully exploited across many tumor types (26, 27). Bevacizumab, aflibercept, ramucirumab, and regorafenib are all FDA approved for use in metastatic colorectal cancer. In addition, bevacizumab is approved for multiple tumor types, including non-small cell lung cancer, ovarian cancer, metastatic renal cell carcinoma (RCC), and glioblastoma. The TKIs have various indications, including the treatment of metastatic RCC, hepatocellular cancer, medullary thyroid cancer, and sarcoma.

The REGARD and RAINBOW studies, published in 2014, led to the approval of ramucirumab in the second line setting in OG cancer (28). Whilst other VEGF targeting drugs (namely bevacizumab and aflibercept) have been investigated in this field, ramucirumab is the only targeted therapy to have FDA and EMA approval in the advanced setting after chemotherapy. There are a number of studies looking in to new combinations of VEGF targeting with both conventional chemotherapy and with immunotherapy. As immunotherapy is likely to be licensed for pre-treated OG cancer this highlights an important area of innovation in this disease. This review aims to outline targeting VEGF in OG cancer, the rationale behind the continued interest in this mechanism and possible future directions in combination with immunotherapy.

## Ramucirumab

Ramucirumab is a fully humanized monoclonal antibody to VEGFR-2, a subtype of the VEGFR which is thought to mediate all known vascular endothelial responses to VEGF (29).

REGARD (30), a randomized phase III placebo controlled trial, investigated ramucirumab in patients who had progressed after first line chemotherapy. One hundred and seventeen patients were randomized to placebo plus BSC and 238 were randomized to ramucirumab plus BSC. Whilst response rates were only 4% with ramucirumab, the rate of stable disease in the treatment arm was 45%, compared with 21% with placebo, giving a disease control rate (DCR) of 45 vs. 21%. Ramucirumab monotherapy increased mOS from 3.8 to 5.2 months [Hazard Ration (HR) 0.776, 95 % CI 0.603–0.998, *p* = 0.047] and median progression free survival (mPFS) from 1.3 to 2.1 months (HR 0.483, 95% CI 0.376–0.620, *p* < 0.0001; Table 1). The treatment was well-tolerated. As expected, rates of hypertension were higher in the treatment than the placebo group (16 vs. 8%) but otherwise there were similar rates of adverse events. This represents a potential treatment option for those patients who are keen to avoid the toxicity of chemotherapy.

**Table 1:** Selected Phase III studies targeting VEGF in OG cancer.

**Trial NCT #**	**Design/setting**	***n***	**Treatment**	**Outcome**	**References**
REGARD NCT00917384	Phase III Randomized Double blind Placebo controlled 2nd line	117	Ramucirumab vs. BSC	OS: R 5.2 vs. BSC 3.8 months (HR 0.776, 95% CI 0.603–0.998, *p* = 0.047) PFS: R 2.1 months vs. BSC 1.3 (HR 0.483, 95% CI 0.376–0.620, *p* < 0.0001) ORR: 4%	(30)
RAINBOW NCT01170663	Phase III Randomized Double blind Placebo controlled 2nd line	335	Paclitaxel ± ramucirumab	OS: P-R 9.6 vs. P 7.4 months (HR 0.807, 95% CI 0.678–0.962, *p* = 0.017) PFS: P-R 4.4 vs. P 2.9 months (HR 0.635, 95% CI 0.536–0.752, *p* < 0.0001). ORR: P-R 28 vs. vs. P 16%	(31)
RAINFALL NCT02314117	Phase III Randomized Double blind Placebo controlled 1st line	616	Capecitabine and cisplatin ± ramucirumab	OS: CX-R 11.17 vs. CX 10.74 months (HR 0.96, 95% CI 0.80–1.16, *p* = 0.68) PFS: CX-R 5.7 months vs. CX 5.4 (HR 0.57, 95% CI 0.61–0.94, *p* = 0.011). ORR: CX-R 41% vs. CX 36% (*p* = 0.17)	(32)
AVAGAST NCT00548548	Phase III Randomized Double blind Placebo controlled 1st line	774	Cisplatin and fluoropyrimidine ± bevacizumab.	OS: FC-B 12.1 months vs. FC 10.1 (HR 0.87, 95% CI 0.73–1.03, *p* = 0/1 PFS: FC-B 6.7 vs. 5.3 months (HR 0.80, 95% CI 0.68–0.93, *p* = 0.004) ORR: FC-B 46% vs. FC 37.4% (*p* = 0.0315)	(33)
NCT01512745	Phase III Randomized Double blind Placebo controlled 3rd line	267	Apatinib vs. placebo	OS: A 6.5 vs. P 4.7 months (HR 0.71, 95% CI 0.54–0.94, *P* < 0.016) PFS: A 2.6 vs. P1.8 months (HR 0.44, 95% CI 0.33–0.61, *P* < 0.001)	(34)

RAINBOW (31), another randomized phase III placebo controlled trial, subsequently investigated combining ramucirumab with paclitaxel in patients with advanced OG adenocarcinoma who had disease progression on or within 4 months of first line chemotherapy. In the study, 335 patients were randomized to paclitaxel with placebo, 330 patients to paclitaxel with ramucirumab. The results demonstrated a significant increase in OS with the combination of ramucirumab with paclitaxel of 9.6 vs. 7.4 months (HR 0.807, 95% CI 0.678–0.962, *p* = 0.017; Table 1). PFS was improved to 4.4 vs. 2.9 months with placebo (HR 0.635, 95% CI 0.536–0.752, *p* < 0.0001). The objective response rate (ORR) was also improved to 28% (vs. 16% with placebo) and the disease control rate was 80% (vs. 64% with placebo). The study reported higher rates of grade 3 or 4 toxicity in the group treated with the combination, although this did not result in higher rates of treatment-related mortality which was 2% in both groups.

In light of the known geographical differences in OG cancer outcomes, the data for RAINBOW was analyzed for Asian and Non-Asian patients as two cohorts. Whilst OS was not significantly improved for Asian patients, the mPFS was. The HRs for OS were 0.73 and 0.99 for non-Asian and Asian patients, respectively and the HRs for PFS were 0.64 and 0.63 for non-Asian and Asian patients. It has been suggested that these differences may be as a result of higher use of third line treatment in Asian populations (almost 70 vs. almost 40%).

A subsequent subgroup analysis of the safety and efficacy of ramucirumab in Japanese and Western patients in RAINBOW (13, 35) noted safety profiles of the ramucirumab plus paclitaxel arm were similar between populations, though there was a higher incidence of grade 3 neutropenia in Japanese patients (66.2 vs. 25.4%). The analysis also reported improved PFS, ORR and 6-month survival rates in the Japanese population compared with the Western population (Table 2). Again post discontinuation therapy rates were much higher in the Japanese than in the Western patients (75 vs. 37%) and it was postulated that this masked any OS benefit.

**Table 2:** Subgroup analysis of RAINBOW efficacy data of ramucirumab in Japanese and Western patients (13).

**Population**	**Overall survival HR**	**PFS HR**	**6 month survival rate %**	**ORR %**
Japanese	0.88 (95% CI 0.60–1.28)	0.50 (95% CI 0.35–0.73)	94.1	41.2
Western	0.73 (95% CI 0.58–0.91)	0.63 (95% CI 0.51–0.79)	66.0	26.8

Following the publication of the REGARD and RAINBOW studies, the FDA granted approval for single agent ramucirumab for the treatment of advanced OG adenocarcinoma that had progressed following 1st line therapy in 2014. Later that year, the FDA then approved the use of ramucirumab combined with paclitaxel to treat advanced OG adenocarcinoma following failure of first line therapy. Ramucirumab has since also been approved by the EMA in both indications although local reimbursement for ramucirumab is variable across different countries.

These studies have validated targeting the VEGF pathway in the 2nd line setting. In the 1st line setting, results have been less encouraging. In 2016, a randomized phase II study failed to show a benefit when adding ramucirumab to FOLFOX in the first line setting for advanced OG adenocarcinoma (36). The multicentre study, involving 168 patients, failed to meet its primary end point of improving PFS [6.4 vs. 6.7 months, HR 0.98 (95% confidence interval 0.69–1.37)]. Objective response rates were also similar between both arms (45.2 vs. 46.4%). The investigators felt that the difference in outcome between this study and REGARD and RAINBOW was likely multifactorial. Firstly, it was postulated that disease biology may be different in 1st and 2nd line settings. Secondly, they noted a higher rate of discontinuation in those treated with ramucirumab and FOLFOX compared to FOLFOX alone. Thirdly, it was noted that this study had a higher proportion of esophageal rather than junctional or gastric tumors than REGARD and RAINBOW and a pre-planned subgroup analysis indicated some benefit in gastric and junctional tumors over esophageal tumors. In gastric, junctional and cardia tumors, mPFS was 8.7 months for the ramucirumab arm vs. 7.1 months in the placebo arm (HR = 0.77) compared to patients with a primary esophageal tumor where mPFS was 5.6 vs. 6.1 months (HR = 1.30).

RAINFALL (NCT02314117), a global phase III study, included 616 patients with advanced gastric, or GOJ adenocarcinoma with tumors of the esophagus excluded. The patients were randomized to either first line treatment with fluoropyrimidine and cisplatin (CX) alone or to CX plus ramucirumab. The study completed in December 2017. The findings revealed a statistically significant 25% reduction in the risk of disease progression or death for the primary endpoint of PFS. PFS was 5.7 months in the intervention arm vs. 5.4 months in the placebo arm (HR 0.75, 95% CI 0.61–0.94, *p* = 0.011). There was no difference in mOS between the ramucirumab and placebo arms (11.17 vs. 10.74 months; HR 0.96, 95% CI 0.80–1.16; *p* = 0.68). There was also no significant difference between ramucirumab and placebo in the ORR (41 vs. 36%; *p* = 0.17) or the DCR (82 vs. 77%; *p* = 0.10) (32). Based on these findings, ramucirumab will not play a role in front line therapy in unselected patients.

The role of ramucirumab in the maintenance setting is currently being explored in the PLATFORM study (NCT02678182). This study will recruit 770 patients to evaluate the efficacy of various maintenance therapies following completion of standard first-line chemotherapy in patients with locally advanced/metastatic HER-2 positive/HER-2 negative OG adenocarcinomas. One of the arms will investigate maintenance capecitabine in combination with ramucirumab.

## Biomarkers and Ramucirumab

Despite the use of anti-angiogenics across multiple indications in cancer there are as yet no robust predictive biomarkers to guide patient selection. Using samples from the REGARD and RAINBOW studies amongst others, attempts have been made to find a predictive biomarker for ramucirumab. Tumor biomarkers such as VEGFR2 and HER2 expression were studied but were not statistically significantly associated with ramucirumab efficacy (37). Serum markers studied include VEGF-C and -D, soluble VEGFR1, 2 and 3, angiopoietin-2, platelet derived growth factor but again baseline levels were not associated with ramucirumab efficacy (37, 38).

Studies have also been conducted in Korean and Japanese patients specifically, investigating trends in potential biomarkers as well as baseline levels. In the Korean study tissue molecular characteristics [Epstein Barr Virus (EBV), Mismatch Repair (MMR), HER2, epidermal growth factor receptor-1 (EGFR-1), hepatocyte growth factor receptor (C-MET) etc.] and circulating biomarkers [VEGF, sVEGFR2, Hepatocyte growth factor (HGF), neuropillin-1, IL-8, and placental growth factor (PIGF)] were assessed. A higher disease control rate with ramucirumab was found in patients with high EGFR expression tumors (2+/3+) compared with low expression tumors (0/1+) (87.5 vs. 50%, *p* = 0.02). A longer PFS was seen in patients with higher level of pre-treatment circulating VEGFR2 (4.1 vs. 2.3 months; *p* = 0.01) and lower level of pre-treatment serum neuropillin-1 (4.1 vs. 2.4 months; *p* = 0.02) (39). The Japanese study focused on dynamic changes in circulating biomarkers. Lower than median Day8/baseline ratios of VEGF-A were significantly associated with a longer PFS (6.3 vs. 2.4 months; *p* = 0.004) and patients with early disease progression had higher Day8/baseline ratios of VEGF-C, Angiopoietin 1, and lower baseline NRP1 levels (40). Both of these studies were small (*n* = 55 and 25, respectively) and these findings require validation but suggest a predictive biomarker for ramucirumab may yet be found.

Evaluation of predictive biomarkers with the use of ramucirumab has also been evaluated in other tumor types. The RAISE study investigated the use of ramucirumab or placebo in combination with FOLFIRI in second line metastatic colorectal cancer and found a significant improvement in OS and PFS with the use of ramucirumab. A subsequent biomarker analysis identified VEGF-D as a potential marker, noting improved median OS in those patients with high levels of VEGF-D compared to low levels and investigators are currently developing an assay for further testing in clinical practice (41).

## Bevacizumab

Bevacizumab is a humanized monoclonal antibody to VEGF-A. This has not demonstrated the same benefit as ramucirumab in OG cancer despite encouraging phase II studies and a proven role in other tumor types. Bevacizumab has been investigated both in the advanced setting and in the peri-operative setting with 2 phase III studies of bevacizumab in the advanced setting, AVAGAST and AVATAR, and one in the peri-operative setting, ST03.

The STO3 study (42) was a multicentre randomized phase II/III trial investigating the addition of bevacizumab to conventional perioperative chemotherapy. Five hundred and thirty three patients received chemotherapy alone and 530 patients received chemotherapy plus bevacizumab. Three-years overall survival was 50% (95% CI 45.5–54.9) in the chemotherapy alone group and 48% (43.2–52.7) in the chemotherapy plus bevacizumab group (HR 1.08, 95% CI 0.91–1.29; *p* = 0.36). With bevacizumab there were increased rates of wound healing complications (12 vs. 7%) and anastamotic leaks in patients who underwent oesophagogastrectomy (24 vs. 10%). The investigators suggested that bevacizumab may have a prolonged effect that delays wound healing. The results of this trial do not support the use of bevacizumab with chemotherapy in the peri-operative setting in unselected patients.

AVAGAST (33) was a global phase III trial to investigate the addition of bevacizumab to 1st line therapy for advanced gastric cancer. Three hundred and eighty seven patients were randomized to doublet therapy of cisplatin with fluoropyrimidine therapy (FC) and 387 patients received FC plus bevacizumab (total 774 patients). Whilst the study did not meet its primary end point with no significant improvement in median OS, 12.1 months with bevacizumab plus FC and 10.1 months with FC alone (HR 0.87, 95% CI 0.73–1.03, *p* = 0.1) there was a trend toward improved survival with bevacizumab. Further, there was a significant improvement in PFS (6.7 vs. 5.3 months, HR 0.80, 95% CI 0.68–0.93, *p* = 0.004) and ORR (46 vs. 37.4%, *p* = 0.0315; Table 1).

Subgroup analysis of AVAGAST revealed that the effect of the addition of bevacizumab varied with geographical location. OS was improved in the pan-America population in comparison to the European and Asian populations (Table 3). The reason for the variability in OS is not clear the authors suggested that it may be as a result of differences in the burden of disease (Asian patients having fewer liver metastases and fewer GOJ tumors) or different patterns of treatment (Asian patients more commonly receive second and further lines of therapy).

**Table 3:** Summary of mOS subgroup analysis in AVAGAST.

**Population**	**FC + Placebo mOS** **(months)**	**FC + Bevacizumab mOS** **(months)**	**Delta** **(months)**	**Hazard ratio**	**95% CI**
Asia	12.1	13.9	1.8	0.97	0.75–1.25
Europe	8.6	11.1	2.5	0.85	0.63–1.14
Pan-America	6.8	11.5	4.7	0.63	0.43–0.94

A pre-planned biomarker analysis following AVAGAST also shed some light on biological differences between the patient populations (43). Markers evaluated included plasma VEGF-A and tumor expression of VEGF-A, VEGFR-1 and−2, neuropilin-1, EGFR-1 and HER2. Plasma VEGF-A levels were higher at baseline in the non-Asian patients whereas neuropilin-1 levels were higher in the Asia-Pacific patients. Both had potential prognostic effects, with high baseline plasma VEGF-A and low tumor neuropilin-1 being associated with worse outcomes. Further, high baseline plasma VEGF-A levels and low tumor neurophilin-1 expression were identified as potential predictive biomarker candidates for bevacizumab efficacy in non-Asian patients although further studies are required to confirm this role. Plasma levels of Angiopoietin-2 (Ang-2) have also been studied in this cohort and again a differential expression was noted between Asian and non-Asian patients. Ang-2 was also associated with a worse OS and the presence of liver metastases but was not predictive for response to bevacizumab and these findings require further validation (44).

As the AVAGAST study only included 12 Chinese patients the AVATAR study was conducted to establish if the geographical effects demonstrated in AVAGAST held true in this population. The study recruited 202 patients who were randomized to capecitabine and cisplatin in combination with bevacizumab or placebo. Again there was no improvement in mOS with bevacizumab and here PFS was similar in both treatment arms (45).

Based on these results it is difficult to see a role for bevacizumab in OG cancer at present, although there are currently phase 1 trials investigating bevacizumab in combination with the anti PDL-1 monocolonal antibody atezolizumab in solid tumors, including esophageal, and gastric cancers (NCT02715531, NCT01633970). Biomarkers of response or resistance remain elusive but bevacizumab's role may be revisited should a robust biomarker be found.

## Aflibercept

Aflibercept is a recombinant fusion protein consisting of human VEGF receptor domains fused with the Fc portion of human Immunoglobulin G (IgG). It binds with circulating VEGF, preventing it from interacting with the VEGFR on endothelial cells and has a high affinity for VEGF-A, VEGF-B and PIGF subtypes. Aflibercept has demonstrated some efficacy in a range of tumor types and is now approved for use in metastatic colorectal cancer in combination with FOLFIRI in the second line setting (46, 47). However, a multicentre randomized phase II trial comparing FOLFOX with either placebo or aflibercept in patients with chemotherapy-naïve metastatic OG adenocarcinoma did not meet its primary endpoint of improved PFS at 6 months (48). Only 64 patients were enrolled and 6 month PFS was found to be 60.5% in the aflibercept arm, compared to 57.1% in the placebo arm (*p* = 0.8) and median PFS was 9.9 vs. 7.3 months, respectively (*p* = 0.69). There are no further on-going studies of aflibercept in OG cancer and it appears unlikely that this drug will be developed further in this setting.

## Multi-Targeted Tyrosine Kinase Inhibitors (TKIs)

Multi-targeted TKIs inhibit angiogenesis via the VEGF pathway and have demonstrated benefit in other tumor types, including GIST, RCC, and NSCLC. Sunitinib, sorafenib, pazopanib, regorafenib, and apatinib have all been investigated in the context of OG cancer either as single agents or in combination with chemotherapy The majority of these studies have been disappointing, with apatinib and regorafenib being the notable exceptions (Table 4).

**Table 4:** Selected studies of TKIs in gastric cancer.

**Trial NCT #**	**Design/setting**	***n***	**Treatment**	**Outcome**	**References**
NCT01187212 STARGATE	Randomized Phase II Open Label Advanced GC	195	Capecitabine and Cisplatin (XP) ± Sorafenib	OS: XP-S 11.7 vs. XP 10.8 months (HR 0.93, 95% CI 0.65–1.31, *p* = 0.661) PFS: XP-S 5.6 vs. XP 5.3 months (HR 0.92, 95% CI 0.67–1.27, *p* = 0.609)	(49)
NCT00226811	Phase II Single arm 2nd line	78	Sunitinib	OS: 6.8 months (95% CI, 4.4–9.6) PFS: 2.3 months (95% CI 1.6–2.6)	(50)
NCT01503372	Phase II Randomized Double blind Placebo controlled 1st line	87	5-FU/Leucovorin/Oxaliplatin (FLO) ± Pazopanib	OS: FLO-P 10.1 vs. FLO 7.0 months (HR 0.80, 95% CI 0.44–1.48) PFS: FLO-P 5.1 vs. FLO 3.9 months (HR 0.93, 95% CI 0.56–1.54)	(51)
ACTRN12612000239864 INTEGRATE	Phase II randomized Double blind Placebo controlled 2nd−3rd line	152	Regorafenib vs. Placebo	OS: Regorafenib 5.8 vs. placebo 4.5 months (HR, 0.74, 95% CI 0.51–1.08, *p* = 0.147) PFS: Regorafenib 2.6 vs. placebo 0.9 months (HR 0.40, 95% CI 0.28–0.59, *p* = <0.001)	(52)
NCT01512745	Phase III Randomized Double blind Placebo controlled 3rd line	267	Apatinib vs. placebo	OS: A 6.5 vs. P 4.7 months (HR 0.71, 95% CI 0.54–0.94, *P* < 0.016) PFS: A 2.6 vs. P 1.8 months (HR 0.44, 95% CI 0.33–0.61, *P* < 0.001)	(34)

As outlined in Table 4, the only TKI to be investigated in phase III trials is apatinib, where an Asian study of 267 patients with advanced gastric or GOJ adenocarcinoma demonstrated a significantly improved median OS with apatinib compared with placebo. It also noted that in this heavily pre-treated population the drug was well-tolerated with an acceptable safety profile (34). Grade 3 to 4 events occurred more frequently in the treatment arm (8.5 vs. 0%) and included hypertension, proteinuria, and neutropenia.

There are three ongoing phase III trials further investigating apatinib in the treatment of advanced OG cancer. Firstly, as the aforementioned phase III trial (34) was in an Asian population, the ANGEL study (NCT03042611), a phase III double blind randomized controlled trial, is investigating apatinib vs. placebo in patients with advanced gastric cancer in Asian as well as European and North American populations. The second is investigating apatinib as maintenance therapy after 1st line chemotherapy (NCT02537171), given that it has been shown to be well-tolerated. The third is investigating the use of apatinib in combination with XELOX chemotherapy as adjuvant treatment for resected gastric cancer (NCT03355612). Given the previous results of bevacizumab in the adjuvant setting in the STO3 study, it will be interesting to see how targeting the VEGF receptor using a different approach fares here.

## Future Combinations with Immunotherapy

Targeting the VEFG pathway in OG adenocarcinoma, through various mechanisms, has been well-investigated with both positive and negative studies as discussed. Given its proven role in the second line setting, there remains a considerable interest in the further development of ramucirumab in OG adenocarcinoma and there are a number studies ongoing. Perhaps the most topical area of study is the combination of ramucirumab and other anti-angiogenics with immunotherapy.

## Immune Checkpoint Blockade in OG Cancer

The use of immune checkpoint inhibitors has brought about a paradigm shift in the treatment of a number of solid tumors and multiple trials of checkpoint inhibitors and other novel drugs targeting various aspects of the immune system are underway in OG cancer. Nivolumab and pembrolizumab have both demonstrated activity in OG cancer when used alone (53, 54), as detailed below. Current studies investigating their combination with ramucirumab are underway. Other immune checkpoint inhibitors investigated in oesophagogastric cancer include avelumab, atezolizumab, durvalumab, and tremelimumab. It is beyond the scope of this article to describe all of these studies but they have recently been well-reviewed by Taieb et al. (55).

Nivolumab, a PD-1 inhibitor, demonstrated a statistically significant improvement in OS in the treatment of advanced chemo-refractory gastric and GOJ cancer in the large phase III ATTRACTION-2 trial involving 493 Asian patients (53). OS was 5.26 months (95% CI 4.60–6.37) in the nivolumab arm and 4.14 months (3.42–4.86) in the placebo arm (hazard ratio 0.63, 95% CI 0.51–0.78; *p* < 0.0001). This study led to a license being granted by the Japanese Ministry of Health, with other regulatory authorities currently reviewing the data.

Nivolumab has also been studied in Western patients in the phase I/II CheckMate-032 study (NCT01928394). Nivolumab was investigated as a single agent and in combination with ipilimumab, a monoclonal antibody against CTLA-4, in the first line setting in patients unselected for PD-L1 status (*n* = 160). Here OS was 6.2 months (95% CI 3.4, 12.4) with nivolumab alone, 6.9 months (95% CI 3.7, 11.5) with nivolumab 1 mg/kg and ipilimumab 3 mg/kg and 4.8 months (95% CI 3.0, 8.4) with nivolumab 3 mg/kg and ipilimumab 1 mg/kg. Using a cut-off of more than or equal to 1% staining for PD-L1 as positive, OS was unchanged in the nivolumab monotherapy cohort and slightly increased in the nivolumab plus ipilimumab cohorts in this group (56). Additional studies of Nivolumab with or without ipilimumab in oesophagogastric cancer are on-going (e.g., NCT02872116, NCT03044613).

Pembrolizumab is another PD-1 inhibitor which has demonstrated activity in gastric and GOJ adenocarcinoma. The KEYNOTE-012 (NCT01848834) phase I trial (*n* = 39) investigated the efficacy of pembrolizumab in patients with advanced solid tumors, including recurrent or metastatic PD-L1 positive gastric cancer (~40% of all gastric cancers). A 22.1% ORR was observed, with 6 month PFS and OS being 24 and 69%, respectively. The authors noted that pembrolizumab demonstrated manageable toxicity and promising anti-tumor activity in this setting (54). Five (13%) patients had grade 3/4 treatment-related adverse events with no treatment related deaths. There were two cases of grade 3 fatigue, one case each of grade 3 pemphigoid, grade 3 hypothyroidism, and grade 3 peripheral sensory neuropathy, and one case of grade 4 pneumonitis.

The subsequent KEYNOTE-059 study (57), a global phase II open-label study, recruited 259 patients with advanced gastric or GOJ cancer who had previously received at least 2 lines of treatment, unselected for PD-L1 status. Single agent pembrolizumab demonstrated promising activity with an ORR of 11.6% in all patients (95% CI, 8.0–16.1%; 30 of 259 patients). The duration of response in these heavily pre-treated patients varied from 1.6 to 17.3+ months (median 8.4 months). Both ORR and duration of response were higher in the PD-L1 positive patients (15.5 vs. 6.4% and 16.3 and 6.9 months, respectively) as was OS, at 5.8 months (95% CI, 4.5–7.9) vs. 4.9 (95% CI, 3.4–6.5) months. Just fewer than 20% patients experienced 1 or more grade 3–5 treatment-related adverse events with 2 patient deaths attributed to treatment.

Based on the KEYNOTE-059 results, the FDA granted accelerated approval to pembrolizumab in Sept 2017 for patients with recurrent locally advanced or metastatic, gastric or GOJ adenocarcinoma whose tumors express PD-L1 as determined by an FDA-approved test. Patients needed to have had disease progression on or after two or more prior specified systemic therapies. This extended the existing tumor agnostic license for pembrolizumab in patients with unresectable or metastatic, microsatellite-instability–high or mismatch-repair–deficient solid tumors, which would apply to ~4–5% gastric tumors. This decision was made as in KEYNOTE-059 55% patients (*n* = 143) had PD-L1 positive tumors and either microsatellite stable (MSS), or undetermined microsatellite instability (MSI) or mismatch repair (MMR) status. In this group the ORR was 13.3% (95% CI: 8.2, 20.0) with over 50% having a response lasting over 6 months and ~25% having a response lasting over a year and these patients would have been ineligible for treatment with the existing license (58).

However, the KEYNOTE-061 study (59) (*n* = 592) has just reported that pembrolizumab did not significantly improve OS in the second line setting for patients with PD-L1 positive oesophagogastric cancer when compared to paclitaxel. Median overall survival was 9·1 months (95% CI 6.2–10.7) with pembrolizumab and 8.3 months (7.6–9.0) with paclitaxel (HR 0.82, 95% CI 0.66–1.03; one-sided *p* = 0.0421) but responses were more durable in the pembrolizumab group than in the paclitaxel group, with a median response duration of 18.0 months (95% CI 8.3–not estimable) vs. 5.2 months (3.2–15.3) and pembrolizumab had a better safety profile than paclitaxel. Additional studies are underway looking at the combination of pembrolizumab with various agents in this disease (e.g., NCT02494583, NCT03382600).

As discussed above, to date a number of studies of checkpoint blockade have provided promising results of activity in OG cancer, although additional randomized trials against chemotherapy are required. Further rationally designed combination studies are also needed to try to maximize the benefit of this approach in appropriately selected patients. The combination of checkpoint blockade and VEGF inhibition is one such option.

## Checkpoint Blockade in Combination With VEGF Inhibition

**Figure 2:**
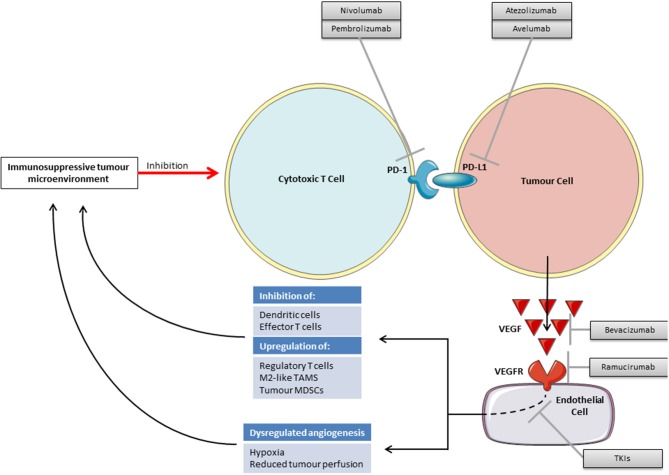
Rationale for combining VEGF inhibition with checkpoint inhibitors.

There is increasing pre-clinical evidence to support VEGF inhibition and immunotherapy as a viable combination strategy. Inhibiting the VEGF pathway may improve the efficacy of checkpoint blockade through both direct effects on the vasculature and through inhibiting VEGF's immunosuppressive functions (Figure 2). There may also be a reciprocal positive impact on the efficacy of anti-angiogenics by vascular changes brought about by immunotherapy.

As reported across multiple tumor types, those patients who respond well to immunotherapy often have an immunologically “hot” tumor containing multiple tumor infiltrating lymphocytes (TILS), whilst those patients with fewer TILs, “cold” tumors, or those with TILS restricted to the margin of the tumor microenvironment (TME), “excluded” tumors, tend to have a lesser response (60–62). For TILs to enter the TME angiogenesis is required to provide blood vessels to deliver them. Cancers are associated with dysregulated angiogenesis, tortuous abnormal blood supplies and resulting hypoxia, high interstitial fluid pressures, and an acid pH (63, 64). Such a hypoxic TME is associated with the recruitment of regulatory T-cells, tumor associated macrophages switching to their immunosuppressive M2 phenotype, a direct inhibition of effector T cells and an accumulation of immunosuppressive metabolites (65).

It may be possible to use anti-angiogenic drugs to normalize tumor vasculature and thereby alleviate this immunosuppressive hypoxia. However, as anti-angiogenic drugs may also destroy blood vessels within tumors rather than normalizing them, this approach will have to be carefully conceived. From animal studies it appears that the effect of anti-VEGF therapy on the vasculature may be dose dependent and as such lower “vascular-normalizing” doses may be required rather than the treatment doses with which we are familiar (66).

In addition to promoting an immunosupportive TME through vascular normalization, anti-VEGF therapy may also reduce direct immunosuppression caused by VEGF. VEGF has various immunosuppressive functions on dendritic cells (DCs), effector T cells, regulatory T cells, myeloid derived suppressor cells (MDSCs) and in facilitating immune evasion through the induction of FAS antigen ligand in endothelial cells and resulting in a barrier to infiltrating CD8 +ve T cells (Table 5) (76). Blockade of VEGF signaling has been shown to reverse these systemic immunosuppressive effects in animal models (65).

**Table 5:** Selected immunosuppressive roles of VEGF.

**Cell type**	**Immunosuppressive impact**	**References**
Dendritic cells	• Inhibition of maturation • Reduction in numbers of DCs	(67) (68)
Effector T cells	• Inhibition of differentiation of progenitor cells into CD4/CD8 +ve T cells • Suppression of proliferation and cytotoxic function • Upregulation of PD-1, CTLA-4, TIM3, and LAG3	(69) (70) (71)
Regulatory T cells	• Increase in number of regulatory T cells	(72) (73)
MDSCs	• Increase in tumor MDSCs	(74) (75)

Preclinically, whilst studies in animal models of OG cancer do not exist, a synergistic effect of VEGF inhibition in combination with immunotherapy has been demonstrated in a number of other tumor types. For example a murine study using Colon-26 adenocarcinoma demonstrated that simultaneous treatment with anti-PD-1 and anti-VEGFR2 monoclonal antibodies resulted in a synergistically increased inhibition of tumor growth compared with either therapy alone without excess toxicity (77). Using a different immunotherapy approach, adoptive T cell transfer, in a mouse model of melanoma the addition of anti-VEGF therapy resulted in significantly increased anti-tumor activity when compared to the immunotherapy alone (78).

A set of experiments with murine models of breast cancer, pancreatic neuroendocrine carcinoma and glioma demonstrated that anti-PD-L1 therapy can sensitize tumors to anti-angiogenic therapy and prolong its efficacy. Further, the experiments also showed the converse, that anti-angiogenic therapy can improve anti-PD-L1 treatment by generating intratumoural high endothelial venules (HEVs) that facilitate enhanced CTL infiltration, activity, and tumor cell destruction in the breast and neuroendocrine but not the glioma models (79).

This combination approach has now been taken forward into clinical trials investigating the use of various anti-angiogenics with immunotherapeutic approaches including checkpoint blockade, vaccination and cell therapies (80). A phase I study of ipilimumab and bevacizumab in patients with advanced melanoma reported a disease control rate of 67% with 24% patients experiencing grade 3/4 toxicity. Tumor biopsies revealed intense infiltration by CD8+ T cells and DCs within the tumor vasculature, with less infiltration seen in those patients treated with ipilimumab alone (81). In colorectal cancer the PD-L1 inhibitor atezolizumab has been investigated in combination with bevacizumab and chemotherapy in a phase Ib study with no unexpected toxicities and a positive signal of activity (82). More advanced studies have reported for RCC and lung cancer.

In RCC a phase II study of bevacizumab and atezolizumab reported encouraging activity in the first line setting in PD-L1 positive patients (83) and the subsequent phase III IMmotion151 study (NCT02420821) study. This study randomized 915 patients with advanced untreated RCC to either a combination of atezolizumab and bevacizumab or sunitinib monotherapy and patients were stratified according to PD-L1 status. The study demonstrated an improved PFS for the combination arm vs. sunitinib in both the intention to treat population [11.2 (95% CI 9.6, 13.3) vs. 8.4 (95% CI 7.5, 9.7) months, HR 0.83, *p* = 0.0219], and the PD-L1 positive population [11.2 (95% CI 8.9, 15) vs. 7.7 (95% CI 6.8, 9.7) months, HR 0.74, *p* = 0.0217]. The combination arm was well-tolerated with a safety profile in keeping with the individual drugs and quality of life was improved, measured as an increased time to interference with activities of daily living in the atezolizumab and bevacizumab combination (11.3 vs. 4.3 months, HR 0.56, 95% CI 0.46, 0.68) (84). Overall survival data were immature and are awaited but this study provides early support for this approach in RCC.

The Phase III IMpower150 study (NCT02366143) assessed the combination of atezolizumab plus carboplatin and paclitaxel with or without bevacizumab vs. carboplatin, paclitaxel, and bevacizumab in patients with advanced Non-Squamous Non-Small Cell Lung Cancer (NSCLC) (85). The addition of atezolizumab to the triplet of carboplatin, paclitaxel and bevacizumab improved OS in the wild type genotype cohort (*n* = 692) from 14.7 to 19.2 months [HR 0.78 (95% CI 0.64, 0.96)]. Grade 3/4 treatment-related adverse events occurred in 55.7% patients with the addition of atezolizumab vs. 47.7% without and were consistent with known toxicity for the drugs involved.

In OG cancer there are several ongoing clinical trials investigating the combination of immune-checkpoint inhibitors with anti-angiogenic therapy (Table 6).

**Table 6:** Selected clinical trials investigating immune-checkpoint inhibitors combined with anti-angiogenic therapy in OG cancer.

**Immune checkpoint inhibitor and anti-angiogenic combination arm**	**Tumor type**	**Study phase**	**Status**	**NCT number**
Pembrolizumab + Ramucirumab	Gastric or gastro-esophageal adenocarcinoma, NSCLC, urothelial carcinoma, or biliary tract cancer	I	Active, not recruiting	NCT02443324 (JVDF)
Durvalumab + Ramucirumab	Gastric or GOJ adenocarcinoma, NSCLC or HCC	I	Active, not recruiting	NCT02572687
Nivolumab + Regorafenib	Gastric cancer, colorectal cancer	I	Recruiting	NCT03406871
Nivolumab + Ramucirumab	Gastric cancer	I/II	Recruiting	NCT02999295
Atezolizumab + Bevacizumab + FOLFOX	Gastric cancer or GOJ	Ib	Recruiting	NCT02715531
SHR-1210 (anti-PD-1 antibody) + Apatinib	Gastric cancer and HCC	I/II	Recruiting	NCT02942329
Pembrolizumab + Lenvatinib	Gastric cancer, breast cancer, ovarian cancer, colorectal cancer, glioblastoma, biliary tract cancers	II	Recruiting	NCT03797326

The JVDF study is a multicentre phase I study of ramucirumab plus pembrolizumab in patients with advanced gastric or GOJ adenocarcinoma, NSCLC, TCC of the urothelium or biliary tract cancer. The trial is split in to 2 phases, the 1st phase determining the safety and tolerability of treatment and the second phase assessing the efficacy of treatment in cohorts of each tumor type. The study is ongoing, however preliminary data from the cohort of patients with advanced gastric or gastro-esophageal junction adenocarcinoma has been presented (86, 87). As of July 2017, 28 treatment naïve OG adenocarcinoma patients had been treated in this study and 68% were PD-L1 positive, assessed by DAKO PD-L1 22C3 IHC pharmDx assay with staining of ≥1% being positive. Treatment-related adverse events occurred in 96% of patients; with 61% experiencing grade 3 adverse events, most commonly hypertension (14%) and diarrhea (11%). No grade 4–5 treatment related events occurred. An objective response was demonstrated in 25% (7/28) of patients with 6 of those responding being positive and 1 negative for PD-L1 expression. The disease control rate was 68%, mPFS 5.3 months (95% CI 3.2–11) and median duration of response was 10 months (95% CI 9.7–10.3). Median OS has not yet been reached (87). These results suggest encouraging activity for the combination in this setting. Activity has also been demonstrated in the second or subsequent line setting in GOJ cancer in another cohort of the JVDF study with a DCR of 46% and a 6 month OS of 51.2% (95% CI, 33.9–66.1) (88).

The phase I clinical trial (NCT02572687) investigating durvalumab, another PD-1 inhibitor, with ramucirumab has also recently had interim results presented. This study enrolled patients with advanced OG adenocarcinoma who had progressed on 1 or 2 lines of systemic therapy. As of May 2017, there were 29 patients in this cohort of whom 48% had PD-L1 ≥25% expression in tumor or immune cells and 3.5% were MSI-high. Seventy two percentage of patients experienced grade 3–4 treatment adverse events. Treatment related adverse events of any grade occurring in over 10% of patients were as expected and included hypertension (34%), fatigue (31%), headache (24%), diarrhea (21%). In this interim analysis 17% of patients achieved a confirmed partial response, including 1 MSI-high patient. For patients with a PD-L1 expression of over 25% the overall response rate was 36%. Progression free survival was 2.6 months (95% CI, 1.45 to 6.28) (89). The final results of this study and JVDF, as well as those detailed in Table 6, are awaited.

## Conclusion

Targeting angiogenesis through the VEGF pathway has been demonstrated to be a viable approach in OG cancer using two different methods, in the form of a monoclonal antibody with ramucirumab and a TKI, apatinib. However, these treatments provide a limited benefit for unselected patients and combination strategies and robust predictive biomarkers are required.

Immune checkpoint blockade has also demonstrated activity in this disease but only for a limited number of patients. Again robust predictive biomarkers are needed as well as methods to convert immunologically “cold or excluded tumors” to “hot tumors” to allow more patients to benefit from this approach.

Combination therapy with anti-angiogenics and immunotherapy may theoretically solve some of these problems, and the scientific rationale is compelling, but a number of hurdles remain. The dose and scheduling of the anti-angiogenic therapy will require careful consideration to ensure the optimum reduction in immunosuppression with vascular normalization, without risking worsening hypoxia or excessive toxicity. A sequencing approach may also be considered.

Biomarkers are required to enable selection of the patients who may respond to each drug and also to inform clinicians as to when optimum vascular normalization has occurred. As discussed biomarkers for anti-angiogenic therapy remain elusive. For immune checkpoint blockade there are multiple biomarkers under investigation, including PD-L1 for which the optimum assay, cut-off, staining pattern, and significance are yet to be established for OG cancer. Other features such as tumor mutational load, microsatellite instability, and an Interferon-γ-related mRNA profile have also been suggested as putative predictive biomarkers for immunotherapy but again additional work is required here (90–93).

Further clarification of the biological differences underlying the geographical variability in response to treatment with anti-angiogenics is also needed to ensure rational drug combinations in different populations. Increased understanding of the microbiome in OG cancer may play a role here, both in understanding geographical differences in treatment response as well as in explaining individual variations in response to immunotherapy. Finally, strategies to overcome resistance, which inevitably develops with targeted therapies and may develop with checkpoint inhibition over time, will be required.

As we further elucidate the role of VEGF and other angiogenic pathways, alongside the immunobiology of OG cancer, it is highly possible that these hurdles will be overcome in this rapidly evolving field and such combinations may become part of the treatment paradigm for this disease in the future.

## Ethics Statement

This article does not contain any studies with human or animal subjects performed by any of the authors.

## Author Contributions

OB and KY contributed equally to this work and wrote the first draft of the manuscript. DC, IC, and NS contributed to manuscript revision, read, and approved the submitted version.

### Conflict of Interest Statement

The authors declare that the research was conducted in the absence of any commercial or financial relationships that could be construed as a potential conflict of interest.
